# The impact of physical activity on working memory in children with ADHD: a meta-analysis

**DOI:** 10.3389/fpsyt.2025.1578614

**Published:** 2025-05-21

**Authors:** Gong Cheng, Ce Song, XiaoQin Hong

**Affiliations:** ^1^ College of Physical Education and Sports, Beijing Normal University, Beijing, China; ^2^ School of Physical Education, Northwest Normal University, Lanzhou, China; ^3^ School of Physical Education, Northern University for Nationalities, Yinchuan, China

**Keywords:** executive function, neurodevelopmental disorder, exercise intervention, cognitive enhancement, attention deficit, systematic review

## Abstract

**Background:**

Attention-Deficit/Hyperactivity Disorder (ADHD) is a common neurodevelopmental disorder in children, often associated with impairments in working memory and other cognitive functions. Physical activity interventions have gained attention as a promising non-pharmacological strategy to alleviate these deficits. The present study aims to systematically evaluate the effects of physical activity on working memory in children with ADHD through meta-analysis, examining its potential mechanisms and providing evidence-based recommendations for comprehensive interventions.

**Methods:**

This systematic review and meta-analysis followed the PRISMA guidelines. PROSPERO registration number CRD420250653800. We included controlled trials involving children clinically diagnosed with ADHD that examined the effects of physical activity interventions on working memory or cognitive functions. Literature was systematically searched in PubMed, Web of Science, Cochrane Library, Embase and CNKI from inception to January 2025. Two independent reviewers screened studies, extracted data, and assessed methodological quality using the Cochrane Risk of Bias Tool. Standardized mean differences were calculated using a random-effects model. Subgroup analyses were conducted by intervention characteristics such as duration, frequency and total time. Sensitivity analyses and publication bias assessment using funnel plots and Egger test were used to evaluate the robustness of the findings.

**Results:**

A total of 11 studies involving 667 participants were included. The meta-analysis showed that physical activity interventions significantly improved working memory in children with ADHD, with a moderate effect size (SMD = 0.51, 95% CI: 0.34 to 0.69). Subgroup analyses indicated that interventions with moderate duration and frequency (45–60 minutes per session, 8–12 weeks, ≤2 times per week, and ≤25 total hours) were associated with the most stable and effective outcomes.

**Conclusion:**

Physical activity interventions are effective in improving working memory in children with ADHD, especially when implemented with optimal session duration, frequency, and total intervention time. These findings support the inclusion of structured physical activity programs in clinical and educational settings.

**Systematic review registration:**

https://www.crd.york.ac.uk/PROSPERO/, identifier CRD420250653800.

## 1 Introduction

Attention Deficit Hyperactivity Disorder (ADHD) is a common neurodevelopmental disorder characterized by inattention, hyperactivity, and impulsive behavior. These symptoms not only affect children’s academic performance but also impact their social skills and daily lives. According to data from the World Health Organization, the global prevalence of ADHD is approximately 5-7.5%, with boys exhibiting a significantly higher incidence than girls (1, 2). This high prevalence makes ADHD a crucial research topic in the field of child mental health. Children with ADHD often display various cognitive deficits, with deficits in working memory being particularly prominent (3). Working memory refers to the ability to store and process information over short periods, which is essential for learning, problem-solving, and task execution (4). Deficits in working memory not only limit the academic achievements of children with ADHD but may also lead to decreased self-esteem, social difficulties, and challenges in future career development (5). These deficits manifest in daily life in various forms, such as difficulty completing homework, forgetting important tasks, and struggling to maintain focus in the classroom, thereby exacerbating their challenges in learning and social environments (6).

There are various intervention methods for children with ADHD, including medication (7), behavioral therapy, and cognitive training (8). Although medication can alleviate symptoms to some extent, it may be associated with side effects and concerns about long-term dependency (9). Behavioral therapy focuses on improving behavior through environmental adjustments and reward systems, but its effectiveness often varies due to individual differences and requires continuous professional support. Cognitive training aims to enhance cognitive functions through specially designed tasks, but the durability and generalizability of its effects remain to be verified (10).In this context, physical activity, as a non-pharmacological and non-invasive intervention, has gradually shown its unique advantages (11). Studies have shown that moderate physical activity can not only improve attention and behavior control in children with ADHD but also significantly enhance working memory (12). Physical activity promotes blood circulation in the brain and neural plasticity, thereby enhancing cognitive functions and improving academic performance and daily living skills (13). Additionally, physical activity is an engaging and easy-to-implement intervention method that can effectively increase children’s participation and sustainability, overcoming many limitations of other intervention methods during implementation (14).

Recent years have witnessed significant advancements in research examining the impact of physical activity on executive functions in children with Attention Deficit Hyperactivity Disorder (ADHD). Through a comprehensive analysis of 22 randomized controlled trials, Huang (15) confirmed that chronic physical activity interventions yield small to moderate positive effects on both core symptoms and executive functions in children with ADHD, with closed-skill exercises (e.g., running, swimming) demonstrating particular efficacy in symptom amelioration. From an alternative perspective, Song (16) conducted a meta-analysis encompassing 24 studies, not only corroborating the beneficial effects of physical activity on inhibitory control, working memory, and cognitive flexibility, but also elucidating the moderating mechanisms of intervention intensity, exercise modality, and frequency in enhancing executive functions. In their theoretical exploration, Welsch (17) proposed the cognitive load hypothesis, suggesting that physically demanding activities with higher cognitive engagement (such as martial arts and ball sports) may offer superior advantages in promoting executive function development. Nevertheless, a critical examination of these studies reveals that while extant literature has established the overall positive impact of physical activity on executive functions in children with ADHD, systematic evaluation regarding the specific mechanisms through which intervention characteristics influence working memory—a core cognitive function—remains inadequate. This research gap constrains our understanding of how physical activity precisely enhances working memory in children with ADHD and impedes the scientific design and implementation of relevant intervention protocols.

Therefore, conducting a systematic meta-analysis is particularly necessary. By integrating existing empirical studies, a meta-analysis can provide more accurate and reliable conclusions, assessing the overall effect of physical activity on enhancing the working memory of children with ADHD. Compared to traditional meta-analyses that focus on executive functions as a whole, conducting a meta-analysis specifically targeting working memory can more precisely reveal the mechanisms through which physical activity affects specific cognitive functions in children with ADHD. This approach provides a scientific basis for developing more effective intervention strategies. In summary, this study aims to systematically evaluate the impact of physical activity on the working memory of children with ADHD through a meta-analysis, exploring its potential mechanisms and application value, with the goal of providing new theoretical support and practical guidance for comprehensive interventions for children with ADHD.

## 2 Research methodology

This study was conducted according to the Preferred Reporting Items for Systematic Reviews and Meta-Analyses (PRISMA 2020) guidelines. PROSPERO registration number CRD420250653800.

### 2.1 Literature search strategy

The search was conducted in both English and Chinese languages, in the following electronic databases: PubMed, Embase (title and abstract), Web of Science, Cochrane Library, and CNKI (title, abstract and keywords), from the inception of each database to January 2025, using the advanced search option. The search strategy combined MeSH terms and free-text keywords to ensure comprehensive coverage, as per standard systematic review practices. The search topics covered three main areas: Attention Deficit Hyperactivity Disorder (ADHD), Physical Activity, and Working Memory. Database-specific filters included human studies, age-appropriate populations, and relevant publication types. The complete search strategy is available in Appendix 1.

### 2.2 Literature inclusion and exclusion criteria

This study defined eligibility criteria following the PICOS (18) framework, with particular emphasis on participant age and intervention characteristics.

#### 2.2.1 Participants

Children and adolescents aged 18 years or younger with a clinical diagnosis of Attention-Deficit/Hyperactivity Disorder (ADHD) based on DSM-5 or ICD criteria.

Intervention (I): Any form of physical activity or exercise-based intervention, including but not limited to aerobic exercise, resistance training, and team sports, with a minimum intervention duration of five weeks.

#### 2.2.2 Comparison

Control conditions included no intervention, routine activities, placebo interventions, or other non-exercise comparators.

#### 2.2.3 Outcomes

The primary outcome was improvement in working memory, assessed using standardized tools such as the Wechsler Memory Scale, n-back tasks, and executive function subscales related to working memory.

#### 2.2.4 Study design

Only randomized controlled trials (RCTs) and controlled non-randomized experimental studies were included.

Exclusion criteria were independently defined to ensure methodological rigor and relevance to the research objective.(1) did not report sufficient methodological details to evaluate study quality (e.g., missing sample size, intervention duration, or outcome measures);(2) involved mixed samples without separate reporting of results for children with ADHD;(3) combined exercise with other interventions (e.g., pharmacological or behavioral therapy) without isolating the effects of physical activity;(4) were not published in peer-reviewed journals (e.g., dissertations, conference abstracts, grey literature);(5) were duplicate publications or secondary analyses of the same dataset.

### 2.3 Literature screening and data extraction

Literature screening and data extraction were conducted strictly following the standard procedures for systematic reviews and meta-analyses, comprising two distinct phases: literature screening and data extraction. Initially, duplicate records were removed using EndNote and Rayyan software. Subsequently, two researchers independently reviewed the titles and abstracts to exclude studies that did not meet the inclusion criteria, such as those not involving children and adolescents with ADHD, interventions that were not physical activities, or studies that did not address working memory outcomes. Studies with ambiguous eligibility were retained for the next phase.After the initial screening, the retained studies underwent full-text evaluation. Each study was assessed against the inclusion and exclusion criteria, and reasons for exclusion (e.g., intervention duration less than five weeks, lack of control group, or incomplete data) were documented. The screening process was independently performed by two researchers, and any disagreements were resolved through discussion or, if necessary, by a third party.

During the data extraction phase, a standardized form was used to collect relevant information, including basic study details (author, year, country), study characteristics (sample size, age range, gender ratio, ADHD diagnostic criteria), intervention characteristics (type of exercise, intensity, frequency, duration, period ≥5 weeks), control measures (no intervention, placebo, or regular activities), outcome indicators (working memory measurement tools and pre- and post-intervention data), and study design (randomization method, allocation concealment). Data extraction was independently carried out by two researchers, followed by cross-verification to ensure accuracy. Discrepancies were resolved through discussion and, if necessary, by a third reviewer. In cases of missing key data, efforts were made to contact the original authors for additional information; if unsuccessful, the studies were documented and excluded. All extracted data were stored in a dedicated database to ensure traceability and provide a reliable foundation for subsequent analyses.

### 2.4 Literature quality evaluation

To evaluate the methodological quality of the included studies, we applied different assessment tools according to study design. For randomized controlled trials (RCTs), the PEDro scale (Physiotherapy Evidence Database) was used (19). PEDro is a widely recognized tool specifically developed for appraising the methodological rigor of intervention studies, particularly RCTs. It includes 11 items covering randomization, allocation concealment, baseline comparability, blinding (participants, therapists, and assessors), outcome follow-up, intention-to-treat analysis, and statistical reporting. Each item (excluding the first) is scored 1 if clearly met, yielding a total score ranging from 0 to 10.

For non-randomized controlled trials, the MINORS (Methodological Index for Non-Randomized Studies) tool was used. This instrument is designed to assess the methodological quality of non-randomized studies, focusing on criteria such as clearly stated aim, inclusion of consecutive participants, prospective data collection, and unbiased assessment of outcomes. Each item is scored from 0 to 2, with higher scores indicating better quality.

All assessments were conducted independently by two reviewers. Disagreements were resolved through discussion or third-party adjudication. The methodological quality ratings were used in sensitivity analyses to evaluate the robustness of the overall findings. Item-level scoring details are provided in the appendix to ensure transparency and reproducibility.

### 2.5 Data analysis

Data analysis was performed using Stata 16.0 software to conduct a meta-analysis of the included studies. The analysis encompassed effect size pooling, subgroup analysis, heterogeneity testing, publication bias assessment, and sensitivity analysis to comprehensively evaluate the impact of physical activity on the working memory of children with ADHD and ensure the robustness and scientific validity of the results.

Effect sizes were calculated based on the method described by Follmann (20), assuming a correlation coefficient of 0.5. The mean differences and standard deviations before and after the intervention were converted into standardized mean differences (SMD) to quantify the intervention effects of physical activity. Due to variations in the working memory assessment tools and indicators across studies, measures were standardized by reversing the direction of means for studies using scales with opposite orientations to ensure consistency.

The SMD and its 95% confidence interval (CI) were used to evaluate the direction and magnitude of the intervention effects, with SMD values of 0.2, 0.5, and 0.8 representing small, medium, and large effects, respectively (21). Heterogeneity was assessed using the I² statistic and Cochran’s Q test. The I² statistic quantified the degree of heterogeneity, while the Q test determined the statistical significance of the heterogeneity. In cases of substantial heterogeneity, potential sources were further explored.

To investigate the potential influence of different intervention conditions on working memory outcomes, detailed subgroup analyses were conducted based on intervention type (e.g., aerobic exercise, strength training), intervention duration (≥5 weeks), intervention frequency, and participant characteristics (e.g., age and gender ratio). Subgroup analyses help identify variations in intervention effects under different conditions and provide a basis for optimizing intervention strategies.

Meta-regression was conducted using R’s metafor package to explore potential moderators of the relationship between physical activity and working memory in children with ADHD. The meta-regression model was specified using the rma() function with restricted maximum likelihood estimation (REML). Model fit was assessed using Q statistics and proportion of variance explained (R²). Significant moderators were identified using p-values, with those below 0.05 considered statistically significant. Regression coefficients and their 95% confidence intervals were reported to indicate the direction and magnitude of moderator effects. This analysis helped identify key factors influencing intervention effectiveness and provided guidance for optimizing physical activity interventions for children with ADHD.

To assess the robustness of the findings, sensitivity analyses were performed by sequentially removing each study from the analysis. If the exclusion of a particular study substantially altered the overall results, the methodological quality and data integrity of that study were re-evaluated. Publication bias was examined using funnel plots, Begg’s test, and Egger’s test. Funnel plots provided a visual inspection of the symmetry in effect sizes across studies. Begg’s test, a rank correlation method, assessed the association between standardized effect sizes and their variances, while Egger’s test evaluated the linear relationship between effect size and precision. A visibly asymmetric funnel plot or a statistically significant result in either test was interpreted as evidence of potential publication bias, indicating the need for cautious interpretation.

### 2.6 Evidence certainty assessment

The certainty of evidence was assessed using the GRADE (Grading of Recommendations, Assessment, Development and Evaluations) approach. This framework evaluates five domains: risk of bias, inconsistency, indirectness, imprecision, and publication bias. For each outcome, the initial level of certainty was determined by the study design, with randomized controlled trials (RCTs) considered high-certainty by default. Two independent reviewers assessed each domain and made judgments about potential downgrading or upgrading based on predefined criteria. Disagreements were resolved by discussion or consultation with a third reviewer. A Summary of Findings table was prepared according to GRADE guidance using the GRADEpro GDT tool.

## 3 Results

### 3.1 Search results

**Figure 1:**
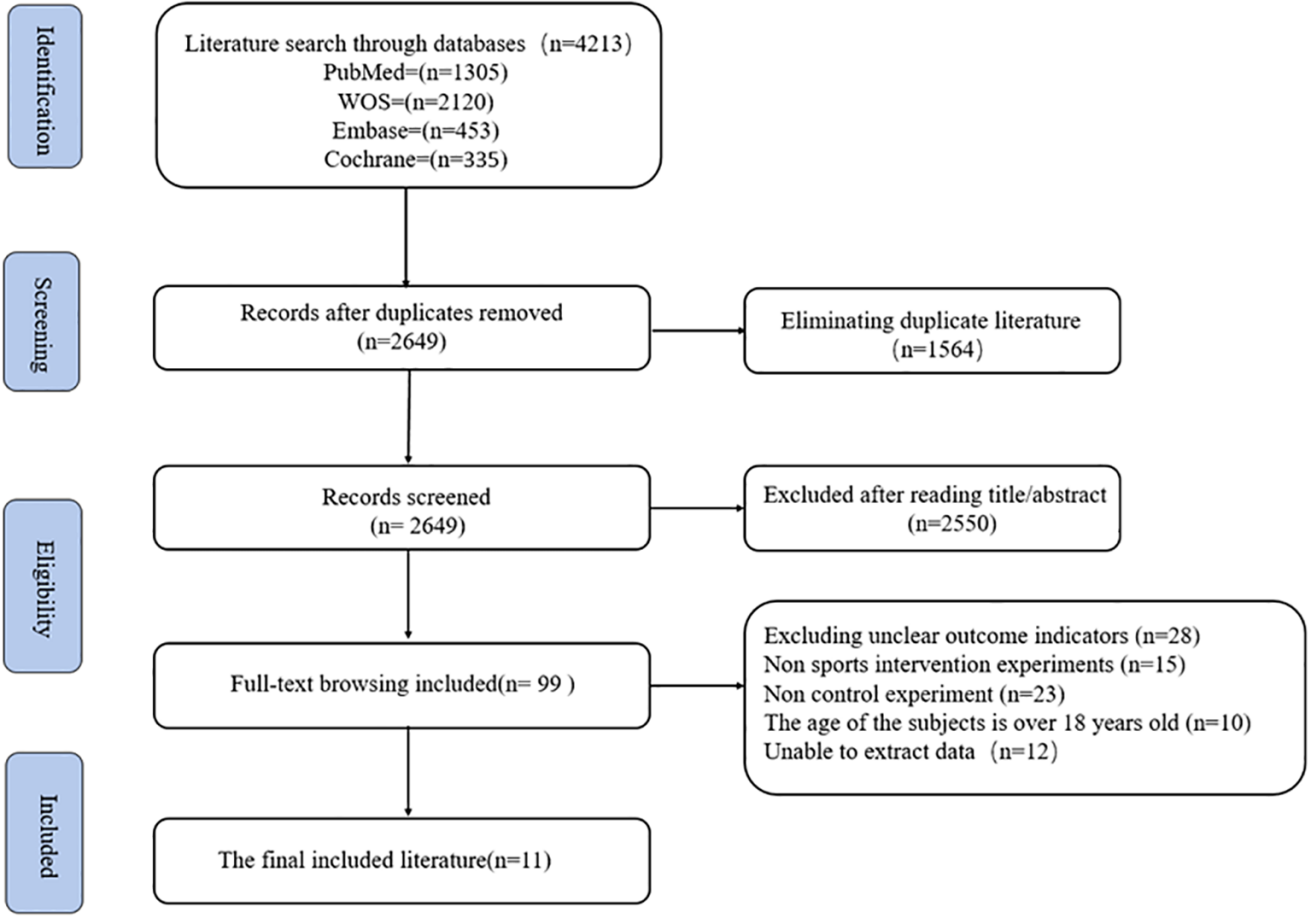
Flowchart of literature screening.

**Figure 1** illustrates the literature search and screening process for the meta-analysis, divided into four stages: identification, screening, eligibility assessment, and final inclusion. Initially, a total of 4,213 articles were retrieved from four databases, including 1,305 from PubMed, 2,120 from Web of Science (WOS), 453 from Embase, and 335 from the Cochrane Library. After removing duplicates, 1,564 articles were excluded, leaving 2,649 articles for title and abstract screening. During this phase, 2,550 irrelevant studies were discarded. In the eligibility assessment stage, full-text reviews were conducted, resulting in the exclusion of 28 studies with unclear outcomes, 15 non-physical activity intervention, 23 Non control experiment, 10 studies with participants older than 18 years, and 12 studies with incomplete data extraction. Ultimately, 11 (22–32) studies met the inclusion criteria and were included in the meta-analysis. See **Figure 1** for details.

### 3.2 Characteristics of included studies

The 11 included studies demonstrated the multifaceted impact of physical activity interventions on the working memory and cognitive functions of children, featuring international diversity, methodological variety, and concentrated sample characteristics. The studies originated from countries such as China, the United States, the Netherlands, Iran, and Germany, reflecting global interest and exploration in this field. The participants’ ages predominantly ranged from 6 to 12 years, with the youngest being 3.6 years and the oldest 13 years. The average age was generally between 7 and 8 years, although some studies did not report the average age but provided age ranges. Regarding sample size, the studies varied significantly, ranging from as few as 14 participants to as many as 112. Smaller sample sizes may affect statistical power, while larger samples enhance the robustness and generalizability of the results.

In terms of intervention measures, the studies exhibited a diverse range of intervention forms, including combined cognitive and aerobic exercise, simple aerobic exercise, judo training, yoga, badminton, and mini-basketball. These physical activity interventions encompassed both individual and team sports, focusing on single aspects of physical activity as well as incorporating elements of cognitive training, thereby reflecting personalized and context-specific designs for the child population. Additionally, there was considerable variation in intervention dosage. For instance, Bustamante (28) implemented a high-intensity intervention of 90 minutes per session, five times per week, over 10 weeks, whereas Pan (25) conducted badminton training for 70 minutes per session, twice per week, over 12 weeks. Overall, the intervention durations ranged from 8 to 15 weeks, and differences in frequency and duration may have varying impacts on the outcomes. See **Table 1** for details.

**Table 1:** Basic information of the included studies.

Study	Country	Sample age (years)	Sample size (E/C)	Experimental group	Intervention dose	Outcome indicator	Diagnostic
Ludyga 2022 (26)	China	Range:8-12 Mean:10.8 ± 1.2	57 (29/28)	Judo Training	60min * 2times * 12weeks	E-Prime	DSM-V
Nejati 2021 (24)	Iran	Range:7-12 Mean:9.43 ± 1.43	30 (15/15)	Combined cognitive and aerobic Exercise	45min * 3times * 5weeks	GNG WCST N-Back	DSM-V
Smith 2020 (22)	USA	Range:5-9 Mean:7.4 ± 1.1	80 (53/27)	Combined cognitive and aerobic Exercise	45min * 3times * 15weeks	CVLT Flanker	DSM-IV
Gelade 2018 (23)	Netherlands	Range:7-13 Mean:9.6 ± 1.67	92 (33/28/31)	Simple Aerobic	45min * 3times * 12weeks	VSWM	DSM-V
Pan 2019 (25)	China	Range:7-12 Mean:9.08 ± 1.43	30 (15/15)	Badminton	70min * 2times * 12weeks	Stroop WCST	DSM-IV
Rezaei 2018 (31)	Iran	Range:7-11 Mean: NA	14 (7/7)	Yoga	45min * 3times * 8weeks	CPT WISC-R	DSM-V
Gelade 2017 (27)	Netherlands	Range:7-13 Mean:9.8 ± 2.0	112 (37/39/36)	Simple Aerobic	45min * 3times * 12weeks	SST VSWM	DSM-IV
Bustamante 2016 (28)	USA	Range:6–12 Mean:9.4 ± 2.2	35 (19/16)	Simple Aerobic	90min * 5times * 10weeks	STOPIT AWMA	DSM-IV
Ziereis 2015 (29)	Germany	Range:7-12 Mean:9.2 ± 1.3	43 (13/14/16)	Combined cognitive and aerobic Exercise	60min * 1times * 12weeks	DSFBT LNST	ICD-10
Liang 2022 (30)	China	Range:6-12 Mean:8.4 ± 1.4	80 (40/40)	Combined cognitive and aerobic Exercise	60min * 3times * 12weeks	ToL Flanker TMT	DSM-V
Liu 2023 (32)	China	Range:3-6 Mean:4.90 ± 0.54	15/15	Mini-basketball	40min * 5times * 12weeks	CHEXI	DSM-V

*Range* refers to the sample age span, while *Mean* represents the average age, expressed as mean ± standard deviation (SD). *NA* indicates that the data is not available or not reported in the study. The outcome indicators include cognitive and memory tasks, such as E-Prime (cognitive testing software), GNG (Go/No-Go task for inhibitory control), WCST (Wisconsin Card Sorting Test for cognitive flexibility), N-Back (working memory task), CVLT (California Verbal Learning Test for memory), and Flanker (response inhibition and attention task). Other tools include VSWM (Visual-Spatial Working Memory), Stroop (cognitive inhibition), CPT (Continuous Performance Test for attention), and STOPIT (response inhibition). Diagnostic criteria include DSM-V (Diagnostic and Statistical Manual of Mental Disorders, 5th Edition), DSM-IV (4th Edition), and ICD-10 (International Classification of Diseases, 10th Edition).

Regarding outcome measures, the studies employed standardized tools for assessing working memory, such as E-Prime, N-Back, Flanker, Stroop, WCST, WISC-R, and TMT. Most studies focused on working memory under different tasks, ensuring multidimensional and scientific evaluation results.

### 3.3 Quality assessment of included studies

The PEDro scale ratings indicated that the overall quality of the included studies was high, with a significant proportion classified as high-quality studies (total score ≥7), including Nejati (24), Gelade (23), Bustamante (28), Ziereis (29), and Gelade (27). Moderate-quality studies (total score 4-6) such as Ludyga (26), Liang (30), and Smith (22) were also present, suggesting that these studies generally met requirements for randomized design, statistical analysis, and result reporting but had shortcomings in allocation concealment and blinding. No low-quality studies (total score <4) were reported. This trend may be attributed to the gradual standardization of intervention study designs and increased emphasis on reporting quality in recent years. For instance, most studies satisfied the PEDro scale requirements for randomization and statistical reporting. However, many studies scored low on items related to blinding (e.g., blinding of participants, therapists, and assessors), likely due to the inherent challenges of implementing blinding in physical activity interventions. Additionally, low scores on “follow-up rates for key outcomes” may reflect common data loss in follow-up studies, exacerbated by prolonged intervention periods and decreased compliance among child participants. These limitations suggest that future research should place greater emphasis on blinding during intervention and assessment processes and improve the completeness of follow-up data to enhance study quality. See **Table 2** for details. In addition, a study by Pan used the MINORS scale to evaluate non-randomized controlled trials. Although this literature was a non-randomized controlled trial, it achieved a relatively high overall MINORS score. See Appendix 1 for details.

**Table 2:** PEDro evaluation scale.

Study	A1	A2	A3	A4	A5	A6	A7	A8	A9	A10	A11	Total score
Ludyga 2022 (26)	1	1	0	1	0	0	0	1	0	1	1	5
Liang 2022 (30)	1	1	0	1	0	0	0	0	0	1	1	4
Nejati 2021 (24)	1	1	2	1	0	0	0	1	1	1	1	8
Smith 2020 (22)	1	1	0	1	0	0	0	1	1	1	1	6
Gelade 2018 (23)	1	1	1	1	0	0	0	1	1	1	1	7
Rezaei 2018 (31)	1	1	0	1	0	0	0	1	1	1	1	6
Gelade 2017 (27)	1	1	0	1	1	0	1	0	1	1	1	7
Bustamante 2016 (28)	1	1	0	1	0	0	1	1	1	1	1	7
Ziereis 2015 (29)	1	1	1	1	0	0	0	1	1	1	1	7
Liu 2023 (32)	1	1	1	1	0	0	0	0	1	1	1	6

A1 refers to the specification of eligibility criteria (not scored). A2–A11 are components of the PEDro scale used to assess methodological quality: A2 (random allocation), A3 (concealed allocation), A4 (baseline group similarity), A5 (participant blinding), A6 (therapist blinding), A7 (assessor blinding), A8 (adequate follow-up >85%), A9 (intention-to-treat analysis), A10 (between-group comparisons), and A11 (reporting of point estimates and variability). The total score is calculated based on A2–A11, reflecting the study’s internal validity and statistical reporting quality.

### 3.4 Analysis results

**Figure 2:**
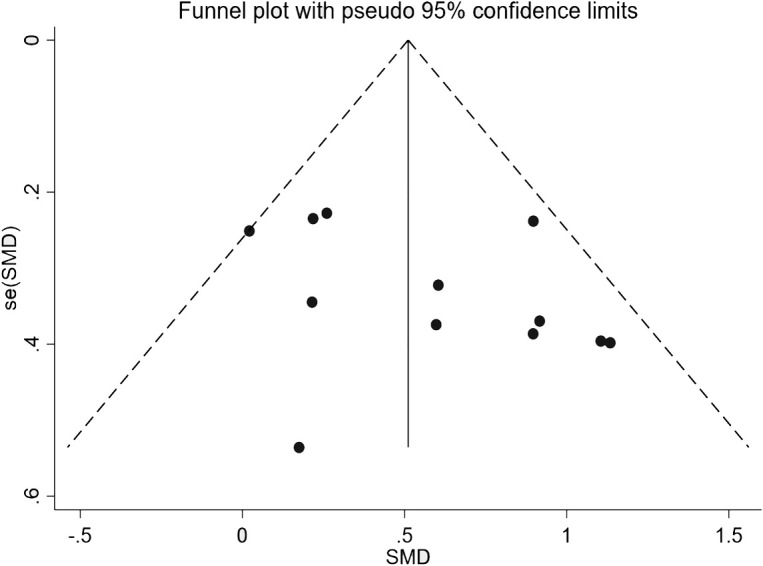
Forest diagram of working memory in attention deficit hyperactivity disorder. Ziereis 2015a and 2015b represent two independent outcomes from the same study, analyzed separately.

The forest plot presents the meta-analysis results of 11 studies on the impact of physical activity on the working memory of children with ADHD. Each study is represented by a black square indicating its effect size, with the size of the square reflecting the study’s weight and the horizontal lines representing the 95% confidence intervals. The blue diamond at the bottom represents the pooled effect size, which is 0.51 (95% CI: 0.34, 0.69), indicating a moderate and significant improvement in working memory among children with ADHD through physical activity interventions. Heterogeneity analysis revealed an I² of 36.7% and a P-value of 0.098, suggesting low to moderate heterogeneity among the studies, thus a fixed-effects model was employed for analysis. See **Figure 2** for details.

Most individual studies reported positive effect sizes, indicating that physical activity interventions improved working memory in children with ADHD. Nejati (24) and Ziereis (29) reported the largest effect sizes of 1.11 (95% CI: 0.33, 1.88) and 1.13 (95% CI: 0.35, 1.92), respectively, both of which were statistically significant (confidence intervals did not cross zero), demonstrating that their intervention programs had a substantial positive impact on working memory. Similarly, Liang (30), Ziereis (29), and Liu (32) reported SMDs of 0.90 (95% CI: 0.43, 1.36), 0.90 (95% CI: 0.14, 1.65), and 0.92 (95% CI: 0.19, 1.64), respectively, all indicating strong positive intervention effects. However, some studies reported lower effect sizes with no statistical significance. For example, Smith (22) reported an SMD of 0.26 (95% CI: -0.19, 0.71), with a confidence interval crossing zero, suggesting limited intervention effects possibly due to small sample sizes, limited intervention efficacy, or participant differences. Additionally, Gelade A (23). reported an SMD of 0.02 (95% CI: -0.47, 0.51), with an effect size near zero and a confidence interval crossing zero, indicating a weak intervention effect. These inconsistencies may be attributed to variations in study design, intervention types and durations, participant characteristics, and measurement tools.

The distribution of study weights varied, with Smith (22) having the highest weight at 15.27%, followed by Gelade (23) at 14.36% and Liang (30) at 13.97%. In contrast, studies with significant effect sizes, such as Nejati (24) and Ziereis (29), had relatively lower weights of 5.05% and 4.99%, respectively. This weight distribution is primarily influenced by sample sizes and data variability, as larger sample sizes and lower variability studies typically receive higher weights in a random-effects model. Overall, the analysis indicates that physical activity has a significant moderate effect on improving the working memory of children with ADHD, providing strong evidence for physical activity as an intervention method. The low heterogeneity among studies suggests high consistency of results. However, the presence of non-significant and highly variable effect sizes in some studies may be related to factors such as differences in intervention types (e.g., aerobic exercise, strength training), intervention durations and frequencies, and participant characteristics like age, gender ratio, and ADHD severity. Future research should aim to standardize intervention designs, control for intervention duration, frequency, and participant characteristics, and increase sample sizes to enhance the precision of results. Additionally, exploring the mechanisms by which different intervention types improve working memory can help identify potential moderating factors, thereby optimizing intervention strategies and reducing heterogeneity among studies.

#### 3.4.1 Subgroup analysis

This study conducted a subgroup analysis to explore the effects of physical activity interventions on the working memory of children with ADHD under different intervention conditions. The subgrouping was based on key intervention characteristics, including session duration (Min), total intervention period (Week), weekly frequency (Times), total intervention time (Allh), and intervention type. This approach provided a descriptive overview of how specific ranges of intervention parameters may relate to intervention efficacy.The results indicated that when each intervention session lasted between 45 and 60 minutes, the effect size was the highest (SMD = 0.86, 95% CI: 0.56, 1.17), and heterogeneity was the lowest (I² = 0.0%). This suggests that moderately extending the duration of each intervention session is beneficial for maximizing improvements in the working memory of children with ADHD. Conversely, shorter durations (N ≤ 45 minutes) or longer durations (60 < N ≤ 90 minutes) resulted in weaker effects (SMDs of 0.34 and 0.39, respectively).In terms of the intervention period, a medium duration (8 < N ≤ 12 weeks) showed better outcomes (SMD = 0.53, 95% CI: 0.34, 0.73). Although a shorter period (N ≤ 8 weeks) had a higher effect size (SMD = 0.78, 95% CI: 0.15, 1.40), it exhibited greater heterogeneity (I² = 48.7%). A longer period (12 < N ≤ 15 weeks) did not show a significant effect (SMD = 0.26, 95% CI: -0.19, 0.71).Analysis of weekly intervention frequency revealed that lower frequencies (N ≤ 2 times per week) had the highest effect size (SMD = 0.78, 95% CI: 0.42, 1.14) with zero heterogeneity, indicating that controlling the intervention frequency moderately helps stabilize the intervention effects. When the frequency increased to 2–3 times or 3–5 times per week, the effect sizes were 0.41 (95% CI: 0.19, 0.62) and 0.54 (95% CI: 0.05, 1.04), respectively, with increased heterogeneity (I² = 54.30% and 48.10%).The total intervention time results showed that short-term, high-efficiency interventions (N ≤ 25 hours) were most effective (SMD = 0.82, 95% CI: 0.48, 1.16) with the lowest heterogeneity (I² = 0.0%). However, as the total intervention time increased (25 < N ≤ 45 hours and 45 < N), the effects gradually weakened, with effect sizes of 0.42 and 0.21, respectively. This suggests that excessively long intervention durations may lead to diminishing returns. See **Table 3** for details.

**Table 3:** Subgroup analysis of working memory.

Overall	Subgroup	SMD (95% CI)	I-squared	p	Number of References
Min	45 < N ≤ 60	0.86 (0.56,1.17)	0.0%	0.768	4
	N ≤ 45	0.34 (0.10,0.57)	39.50%	0.142	6
	60 < N ≤ 90	0.39 (-0.11,0.89)	0.0%	0.452	2
Week	8 < N ≤ 12	0.53 (0.34,0.73)	40.60%	0.097	9
	N ≤ 8	0.78 (0.15,1.40)	48.7%,	0.163	2
	12 < N ≤ 15	0.26 (-0.19,0.71)	–	–	1
Times	N ≤ 2	0.78 (0.42,1.14)	0.0%	0.702	4
	2 < N ≤ 3	0.41 (0.19,0.62)	54.30%	0.052	6
	3 < N ≤ 5	0.54 (0.05,1.04)	48.10%	0.165	2
Allh	N ≤ 25	0.82 (0.48,1.16)	0.0%	0.545	5
	25 < N ≤ 45	0.42 (0.21,0.64)	48.90%	0.081	6
	45 < N	0.21 (-0.46,0.89)	–	–	1
Intervention	Judo Training	0.60 (-0.03,1.24)	–	–	1
	Combined cognitive and aerobic Exercise	0.82 (0.51,1.14)	30.70%	0.228	4
	Simple Aerobic	0.33 (0.04,0.61)	0.00%	0.466	4
	Badminton	0.02 (-0.47,0.51)	–	–	1
	Yoga	0.60 (-0.14,1.33)	–	–	1
	Mini-basketball	0.92 (0.19,1.64)	–	–	1

Min refers to the duration of each intervention session (in minutes); Week indicates the intervention period (in weeks); Times represents the frequency of intervention per week; Allh denotes the total intervention hours.

The effects of different types of physical activity interventions on working memory in children with ADHD were further explored within the subgroup analysis. The results indicated that the Mini-basketball intervention yielded the highest effect size (SMD = 0.92, 95% CI: 0.19 to 1.64), suggesting a potentially significant advantage in enhancing working memory. Combined cognitive and aerobic also demonstrated a relatively high effect size (SMD = 0.82, 95% CI: 0.51 to 1.14), with low heterogeneity (I² = 30.7%), indicating a more stable intervention effect. Judo Training and Yoga both showed moderate effect sizes (SMD = 0.60, 95% CI: -0.03 to 1.24 and -0.14 to 1.33, respectively), suggesting some potential benefits; however, the confidence intervals crossing zero imply that the stability of these effects requires further verification. Simple Aerobic interventions had a smaller but statistically significant effect size (SMD = 0.33, 95% CI: 0.04 to 0.61), with no observed heterogeneity (I² = 0.0%), indicating good consistency in the results. In contrast, the Badminton group showed a negligible effect (SMD = 0.02, 95% CI: -0.47 to 0.51).

#### 3.4.2 Meta-regression analysis for moderator effects

To further explore the potential sources of heterogeneity and quantitatively assess the influence of intervention characteristics on effect sizes, a meta-regression analysis was conducted. The analysis incorporated four continuous variables: intervention duration per session (minutes), total intervention period (weeks), weekly frequency (times/week), and total intervention time (hours). The results revealed that longer durations per session were significantly associated with greater improvements in working memory (β = 0.018, p = 0.024), supporting the subgroup finding that 45–60-minute sessions yielded the largest effects. Conversely, total intervention time showed a significant negative association with effect sizes (β = -0.027, p = 0.046), suggesting that excessively prolonged interventions may lead to diminishing returns. Weekly frequency (β = 0.175, p = 0.232) and overall intervention duration (β = -0.011, p = 0.747) did not show significant associations, implying that dose quality may matter more than quantity alone. Additionally, a separate meta-regression model was employed to examine the impact of intervention types. The results indicated that both ball sports (β= 0.759, p = 0.012) and combined cognitive and aerobic (β= 0.754, p < 0.001) had significantly positive effects on working memory, aligning with previous subgroup analyses. Other types, such as mind–body interventions (β= 0.471, p = 0.142) and simple aerobic activities (β= 0.274, p = 0.132), showed positive but non-significant trends. However, given the limited number of studies included in each moderator category, these findings should be interpreted with caution and warrant further verification through larger-scale primary studies. See Appendix 1 for details.

#### 3.4.3 Sensitivity analysis

**Figure 3:**
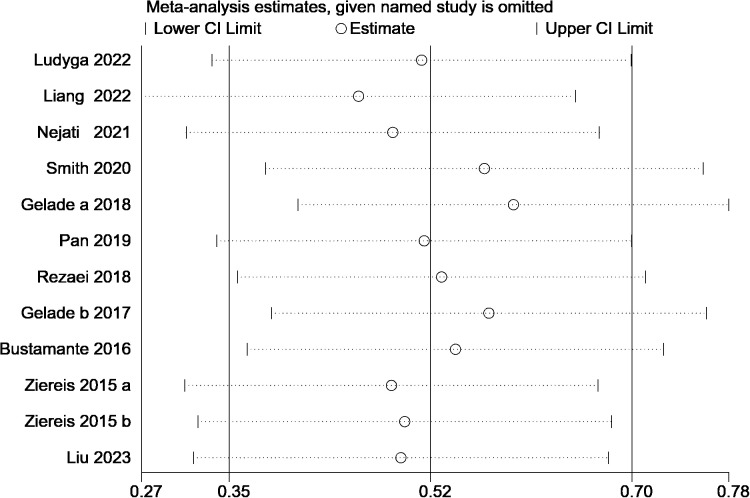
Leave-one-out sensitivity analysis plot.

The sensitivity analysis plot displays the changes in the overall effect size and its 95% confidence interval after excluding individual studies. For example, after excluding Ludyga (26), the point estimate of the overall effect size slightly increased, and the 95% confidence interval remained narrow, indicating that this study had a limited impact on the overall results. In contrast, excluding Nejati (24) caused a slight decrease in the effect size point estimate, and the lower limit of the confidence interval shifted slightly to the left. However, the magnitude of these changes remained within an acceptable range and did not substantially affect the results. See **Figure 3** for details.

Overall, excluding any single study did not lead to a significant shift in the overall effect size, and the range of the 95% confidence interval remained within a reasonable scope without any abnormal widening or narrowing. This suggests that the results of all individual studies are balanced in the overall meta-analysis, and no single study has an excessive influence on the overall outcome. The sensitivity analysis results further confirm the robustness and reliability of this meta-analysis, indicating that the final combined effect size conclusion is highly stable, trustworthy, and not easily influenced by any single study.

#### 3.4.4 Publication bias

**Figure 4:**
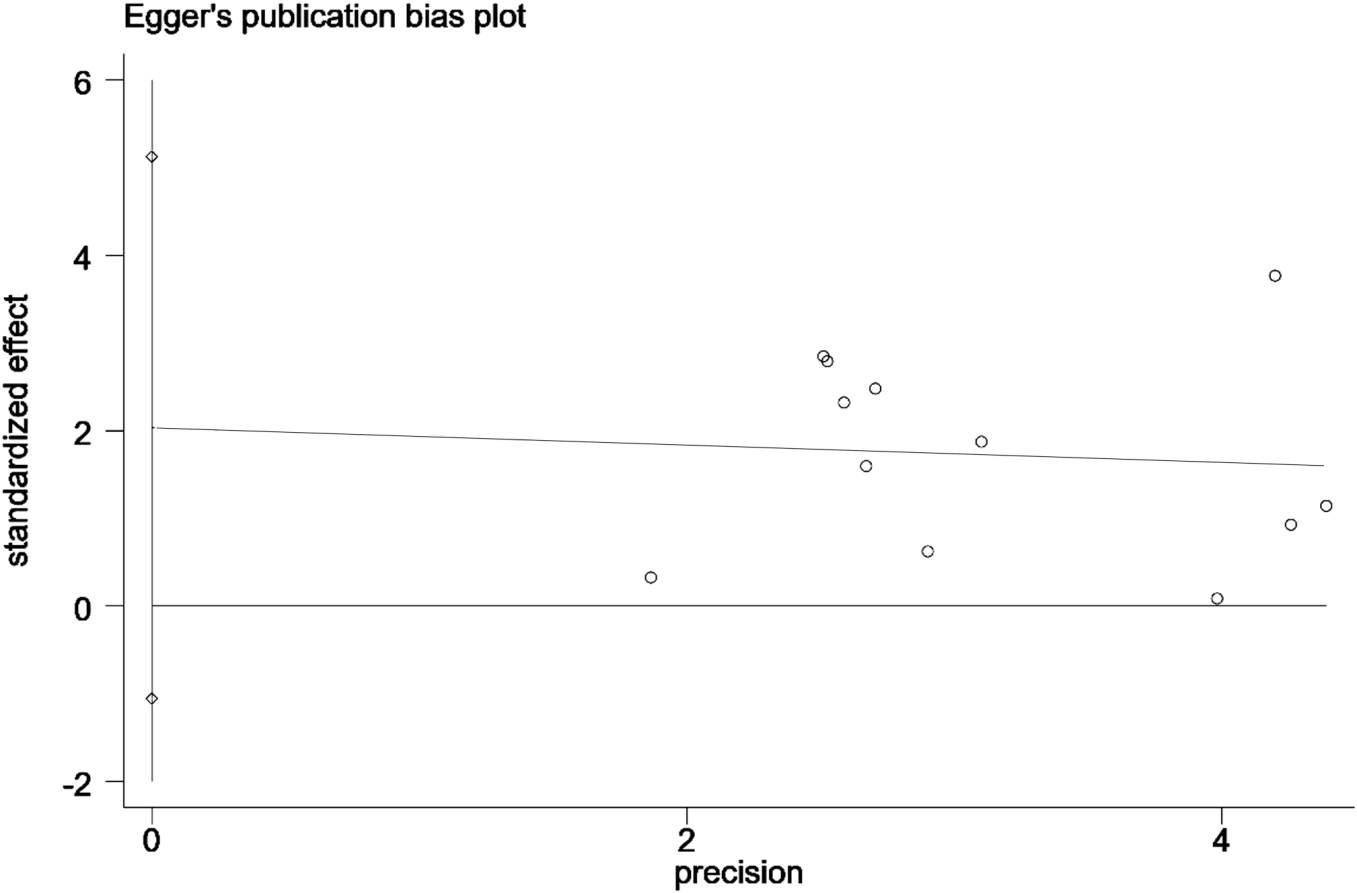
Publication bias funnel plot.

Based on the results of Begg’s test and Egger’s test, no significant publication bias was detected. In Begg’s test, the Pr > |z| values were 0.131 and 0.150 (with continuity correction), and in Egger’s test, the P>|t| value for the bias term was 0.173. All values were greater than the significance level of 0.05, indicating that the statistical tests did not detect the presence of publication bias. Additionally, the funnel plot showed that the study points were roughly symmetrically distributed on both sides of the overall effect, with most points falling within the 95% confidence interval. This indicates that no obvious asymmetry was observed, further supporting the conclusion that publication bias does not exist. See **Figure 4** for details.

**Figure 5:**
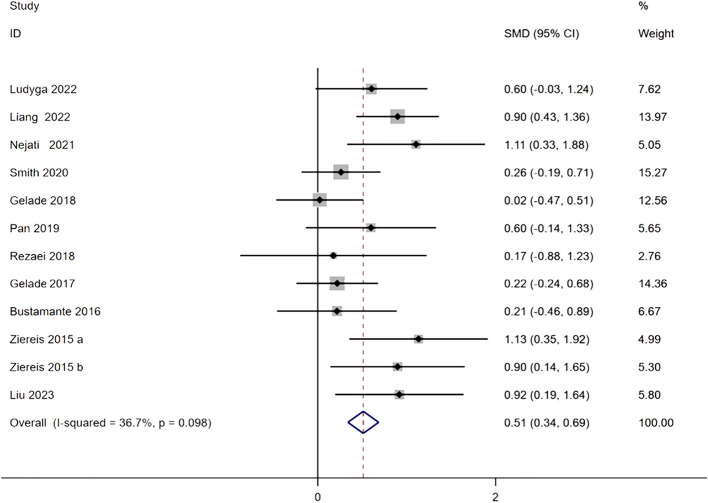
Egger linear regression plot.

Combined with Egger’s publication bias plot, the scatter plot of standardized effect sizes versus precision showed a gentle linear trend, and the regression line (red line) was close to horizontal, showing no significant deviation. This further corroborates the low risk of publication bias. Therefore, considering the results of the statistical tests, funnel plot, and Egger’s regression plot, it can be concluded that the risk of publication bias in the current analysis is minimal, and the results are stable and reliable. See **Figure 5** for details.

#### 3.4.5 Results of evidence certainty assessment

The certainty of the evidence for the primary outcome, working memory improvement, was assessed using the GRADE framework. Based on 11 randomized controlled trials involving a total of 603 participants (357 in the intervention group and 246 in the control group), the overall certainty was rated as moderate. Downgrading was applied due to serious risk of bias, as several studies lacked allocation concealment and participant or assessor blinding. Additionally, imprecision was noted, as some studies had small sample sizes and wide confidence intervals. No serious inconsistency was observed (I² = 36. 7%), and sensitivity analysis confirmed the robustness of the pooled results. Publication bias was not detected, as indicated by symmetrical funnel plots and non-significant Egger’s and Begg’s test results (p > 0. 05). The Summary of Findings table presents the detailed GRADE assessment. The detailed GRADE assessment is presented in Appendix 1.

## 4 Discussion

This study systematically evaluated the impact of physical activity interventions on the working memory and cognitive functions of children with ADHD through a meta-analysis. The participants were primarily aged between 6 and 12 years. The overall analysis revealed that physical activity significantly improved the working memory of children with ADHD with a moderate effect size (SMD = 0.51, 95% CI: 0.34, 0.69). Further subgroup analysis indicated that moderate intervention conditions—such as session durations of 45–60 minutes, intervention periods of 8–12 weeks, weekly intervention frequencies of ≤2 times, and total intervention durations of ≤25 hours—maximized improvements in working memory performance. Additionally, the quality assessment of the literature indicated that the included studies were generally of high quality, ensuring the reliability and robustness of the meta-analysis results. These results may be influenced by various factors, including participant compliance, individual differences, and recovery time. Longer durations or higher frequencies of intervention might cause fatigue, reduced compliance, or marginal decreases in effectiveness. The advantage of this subgroup analysis lies in its comprehensive examination of intervention duration, period, frequency, and total time. Through stratified analysis, it reveals differences in effects under various intervention conditions, providing empirical evidence for optimizing clinical practices and intervention plans.

While the subgroup analysis offered useful descriptive insights into the potential influence of different intervention characteristics, it did not directly test the statistical relationships between these factors and the observed effect sizes. To address this limitation, we conducted a meta-regression analysis incorporating the same factors as continuous predictors. This allowed for a more formal examination of their association with intervention outcomes. The regression results confirmed that session duration and total intervention time significantly influenced the intervention effects, thereby validating the key patterns observed in the subgroup analysis. Team-based ball games such as Mini-basketball typically require participants to engage in continuous rapid responses, spatial judgment, and strategic decision-making. These activities are likely to activate multiple brain regions associated with executive functions, thereby significantly enhancing attentional control and working memory. Combined cognitive and aerobic exercise, which integrate physical activity with cognitive tasks—such as counting, memorization, or decision-making—consistently stimulate brain regions involved in cognitive processing during exercise, potentially promoting neuroplasticity and improvements in working memory. Judo Training, which combines rule learning, self-regulation, and motor coordination, may be particularly beneficial for improving impulse control and attention in children with ADHD, and holds promise for demonstrating stronger effects in future research. Although both Yoga and Judo Training showed moderate effect sizes, their emphasis on breath regulation, self-awareness, and mental focus may confer unique benefits for enhancing attentional stability and emotional self-regulation. These qualities could be especially valuable for individuals with ADHD who exhibit emotional lability and fluctuating attention. By contrast, simple aerobic exercises, such as running or calisthenics, involve lower levels of cognitive engagement, which may limit their efficacy in enhancing executive functions and working memory. In addition, some intervention measures, including Badminton, Judo Training, Yoga, and Mini-basketball, were supported by only a single study with relatively small sample sizes. Therefore, definitive conclusions about their efficacy remain premature. Future research should prioritize high-quality randomized controlled trials targeting specific forms of physical activity to elucidate their underlying mechanisms and determine their suitability for different subgroups of children with ADHD.

A considerable number of interventional studies incorporated adaptive modifications throughout the intervention process to address the unique characteristics of children with ADHD. For example, Smith (22). IBBS intervention incorporated dynamically adjusted game difficulty based on participants’ performance, as well as immediate feedback and a reward system (e.g., point-based incentives), which effectively enhanced task engagement and motivation. This reflects a highly individualized, child-centered design strategy. Similarly, Nejati (24). and Rezaei (31). considered the cognitive characteristics of children with ADHD by implementing measures such as sequencing cognitive tasks to mitigate fatigue, incorporating short-interval practice to sustain attention, and providing real-time feedback in neurofeedback training—each representing targeted, function-specific adaptations. Some studies, such as those by Pan (25). and Liang (30), did not explicitly report ADHD-specific adjustments, but their intervention content nonetheless addressed core cognitive and behavioral challenges. Approaches such as multimodal integration (e.g., combining cognitive training with physical activity) and tiered task complexity implicitly supported improvements in attention, executive functioning, and motor control. While designing a semi-active control condition, Gelade (23) simultaneously implemented precise regulation of training intensity and heart rate, demonstrating a focus on physiological adaptability.

In contrast, studies such as those by Ziereis (29). and Ludyga (26). mentioned procedural steps like coach training or familiarization with the testing environment but offered limited detail regarding adaptations for ADHD-specific behavioral or cognitive profiles. As a result, the extent of adaptive intervention design remains unclear in these cases. These differences highlight the need for future research to systematically document individualized and dynamic adjustment strategies—particularly with respect to task difficulty calibration, attentional engagement, and feedback mechanisms—to better tailor interventions for children with ADHD.

The findings of this study are highly consistent with existing cognitive neuroscience theories, particularly echoing the executive function theory and the neuroplasticity theory (33). The executive function theory posits that working memory is a core component of executive functions, and physical activity interventions enhance working memory capacity by increasing activity in the prefrontal cortex (34, 35). Significant effects observed in studies by Nejati (24) and Ziereis (29) support the positive impact of exercise on cognitive functions, especially working memory, aligning with previous research findings by Smith (22) and Liang (30). However, some studies, such as those by Smith (22) and Gelade (23), did not show significant effects, which may reflect the influence of different intervention types or research designs on result consistency. This suggests that the applicability of the theory needs further exploration in various contexts.

The improvement in working memory of children with ADHD through physical activity interventions is primarily achieved via neurotransmitter regulation and neuroplasticity mechanisms (36). At the neurotransmitter level, physical activity significantly modulates the levels of dopamine and norepinephrine in the brain (37, 38). Under the regulation of the prefrontal cortex, these neurotransmitters enhance the efficiency of signal transmission between neurons, thereby improving information processing and working memory capacity. Dopamine optimizes neuronal excitability by binding to receptors, while the release of norepinephrine further enhances attention focus and reaction speed (39). Concurrently, exercise significantly increases blood flow to the prefrontal cortex, providing neurons with sufficient oxygen and glucose to support their efficient functioning. This increase in blood flow not only improves neuronal energy metabolism but also facilitates the clearance of metabolic waste, maintaining the stability of neural networks. At the molecular level, exercise promotes the expression of brain-derived neurotrophic factor (BDNF) (40, 41). By binding to TrkB receptors, BDNF activates downstream signaling pathways such as PI3K/Akt and MAPK/ERK, promoting neuronal growth, differentiation, and synaptic plasticity, thereby optimizing information transmission and storage capabilities (42, 43).

Physical activity interventions regulate the hypothalamic-pituitary-adrenal (HPA) axis activity (44), effectively reducing levels of stress hormones like cortisol and alleviating the neural stress burden (45). This reduction in stress levels not only contributes to emotional stability but also decreases cognitive load, enabling children with ADHD to better concentrate on information processing (46). In terms of energy metabolism, exercise enhances mitochondrial function in neurons, increasing intracellular energy metabolism efficiency (47). As the energy factories of cells, enhanced mitochondrial function ensures that neurons receive adequate ATP supply, supporting efficient electrical signal transmission and synaptic activity, and reducing neuronal fatigue and dysfunction caused by energy deficits. Additionally, the anti-inflammatory effects of exercise, through the reduction of inflammatory cytokines such as TNF-α and IL-6, lower neuroinflammation levels, protect neurons from inflammatory damage, and promote neural repair and regeneration. This further supports the recovery and enhancement of cognitive functions (48).

The effectiveness of physical activity interventions is also closely related to their implementation methods and microstructural changes, reflecting the systematic and complex nature of the intervention process. Moderate intervention intensity and reasonable frequency play coordinating and balancing roles in these mechanisms, ensuring the effective release of neurotransmitters and BDNF while avoiding fatigue and stress responses caused by overloading (49). On a microstructural level, exercise promotes an increase in synaptic density and the expansion of postsynaptic densities, making connections between neurons tighter and more efficient, thereby enhancing the speed and accuracy of information transmission (50). These structural changes collectively improve the capacity and processing speed of working memory, enabling the brain to more flexibly handle complex cognitive tasks. Different forms of physical activities, such as team sports and aerobic exercises, further enhance cognitive functions by promoting social interactions and emotional regulation (50). It is noteworthy that future research should further explore the specific impacts of different types of exercises on these neurochemical and structural changes, clarifying the causal relationships and interactions among various mechanisms to provide more detailed scientific evidence for designing personalized intervention programs. Additionally, considering the potential response differences among children of different age groups, genders, and ADHD severity levels, exploring more segmented and targeted intervention strategies is particularly important.

### 4.1 Limitations and future directions

Although this study provides important insights into the impact of physical activity interventions on the working memory of children with ADHD, several limitations must be acknowledged and discussed. The number of included studies was relatively limited, with only 11 studies meeting the inclusion criteria, which may affect the generalizability and statistical power of the meta-analysis results. Particularly in subgroup analyses, some groups included only single studies, leading to unstable effect size estimates and potential bias. Additionally, the included studies exhibited considerable heterogeneity in terms of intervention types, periods, frequencies, and durations. Although the overall heterogeneity was low (I² = 36.7%), certain subgroups displayed high heterogeneity (e.g., weekly intervention frequencies of 2–3 times and 3–5 times), suggesting that different study designs and intervention protocols might influence the results. Additionally, many included studies did not clearly report or standardize exercise intensity, which might significantly influence cognitive outcomes. Variations or uncertainties in exercise intensity could contribute to the observed heterogeneity and limit precise interpretation of the effectiveness of specific physical activity interventions.

Moreover, this meta-analysis did not provide a refined classification of specific sports disciplines, Because many interventions were inherently composed of mixed or combined exercise types, specific exercise modalities were not clearly distinguished or explicitly described. While a few interventions—such as judo, yoga, badminton, and mini-basketball—were clearly defined, many studies employed broad categories such as “simple aerobic” and “Combined cognitive and aerobic Exercise.” “Simple aerobic” generally refers to repeated, low-complexity exercises like running or basic aerobic routines, whereas “Combined cognitive and aerobic Exercise” is characterized by the addition of cognitive challenges (e.g., memory, attention, or executive function tasks) embedded within physical movement. it is important to note that “Combined cognitive and aerobic Exercise” itself is not a single, standardized exercise modality, but rather a combined intervention format involving multiple types of physical activities interwoven with cognitive elements. Due to this variability, and the lack of detailed reporting in many studies, it remains unclear which specific physical or cognitive components drive the observed effects. Similarly, some interventions labeled under the same category may in fact differ substantially in both structure and intensity. This imprecise classification limits our ability to compare across studies and draw conclusions about the relative efficacy of different exercise types. Future research should aim to define and report intervention content more specifically—clearly distinguishing between types of movements, cognitive demands, and sports disciplines—to improve the interpretability and practical relevance of findings.

## 5 Conclusion

This study conducted a systematic meta-analysis to evaluate the effects of physical activity interventions on the working memory and cognitive functions of children with ADHD. The findings indicate that physical activity significantly improves the working memory of children with ADHD, with moderate-intensity interventions showing the most pronounced effects. Subgroup analysis further revealed that intervention duration, period, frequency, and total time play regulatory roles in the effectiveness, highlighting the importance of optimizing intervention designs. Although this study provides valuable insights, the interpretation and application of its results should be approached with caution, particularly regarding the diversity in sample sizes and intervention protocols.

## Data Availability

To obtain raw data please contact the author directly by email. Requests to access these datasets should be directed to Chenggong, chenggong202512@outlook.com.
